# Magnetic Biocomposite Based on Aspen Biochar, Sodium Alginate, and *Phaffia rhodozyma* Yeast for Efficient Removal of Methylene Blue from Aqueous Solutions

**DOI:** 10.3390/ma19091894

**Published:** 2026-05-04

**Authors:** Paweł Staroń, Gabriela Gaik, Jarosław Chwastowski

**Affiliations:** Faculty of Chemical Engineering and Technology, Cracow University of Technology, 24 Warszawska St., 31-155 Cracow, Polandjaroslaw.chwastowski@pk.edu.pl (J.C.)

**Keywords:** adsorption isotherms, cationic dye, wastewater treatment, regeneration and reuse, superparamagnetism

## Abstract

The aim of this study was to produce and characterize a magnetic biocomposite based on aspen biochar, sodium alginate, and *Phaffia rhodozyma* yeast biomass, as well as to evaluate its suitability for removing methylene blue (MB) from aqueous solutions. The sorbent structure was confirmed by FTIR, XRD, and SEM, demonstrating successful immobilization of biotic components in an amorphous polymer matrix. Kinetic studies demonstrated a rapid process, with dynamic equilibrium established after 180 min. Experimental data from equilibrium studies (3 h and 24 h) were analyzed using the Langmuir, Freundlich, Temkin, and Dubinin–Radushkevich models. The theoretical maximum sorption capacity (q_d_) determined was 39.31 mg/g, with higher sorption values observed for 24 h confirming the contribution of intrapore diffusion and yeast biosorption activity. In temperature-effect studies, the highest process efficiency (q_e_ = 1.43 mg/g) was observed at 25 °C, while its decrease at 35 °C indicated the exothermic nature of the phenomenon and the thermal sensitivity of the biological structure. VSM analysis revealed superparamagnetic properties of the composite (Ms = 9.3 A·m^2^/kg), which enabled full phase separation. Regeneration studies demonstrated that despite the high efficiency of mineral acids, the use of ethanol as an eluent allows for maintaining the structural integrity of the sorbent and its effective use in at least four cycles. The results indicate that the developed biocomposite is a promising, low-cost, and easily recoverable alternative to conventional sorbents in industrial wastewater treatment technologies.

## 1. Introduction

The increasing release of synthetic dyes into the aquatic environment has become a serious environmental concern due to their persistence, toxicity, and resistance to conventional wastewater treatment methods. Among the various dye contaminants, methylene blue is one of the most commonly used cationic dyes in the textile, paper, and pharmaceutical industries. Although widely used due to its stability and strong coloring properties, its presence in wastewater can lead to adverse ecological and health effects. In addition, methylene blue is frequently used as a model cationic dye in adsorption studies because its high stability and strong absorbance (λ = 664 nm) enable accurate quantification and facilitate comparison with the literature data. Therefore, developing efficient, sustainable, and economically viable methods for removing dyes from aqueous solutions remains a significant research challenge [1,2,3].

Adsorption is considered one of the most effective techniques for removing organic pollutants from water due to its simplicity, high efficiency, and the ability to use inexpensive materials as adsorbents. In recent years, biochar-derived materials have gained significant popularity as promising adsorbents due to their high surface area, porous structure, and the presence of various functional groups capable of interacting with contaminants [4]. Biochar produced by the pyrolysis of lignocellulosic biomass is an environmentally friendly and renewable material that can be obtained from commonly available waste. However, conventional biochars often suffer from limitations related to their recovery from aqueous solutions, and in some cases, insufficient adsorption capacity [5,6]. To overcome these drawbacks, increasing attention is being paid to the development of magnetic biochar-based composites [7]. The introduction of magnetic nanoparticles, such as iron oxides, enables rapid and efficient separation of the adsorbent from purified water using an external magnetic field. This property significantly increases the practical application of such materials in water treatment technologies [8,9].

Another promising approach to improving adsorption efficiency is to incorporate biological components into the adsorbent structure. Microorganisms, including bacteria, fungi, and yeasts, possess cell walls rich in functional groups such as hydroxyl, carboxyl, amine, and phosphate groups, which can actively participate in contaminant binding. Biosorption processes based on bacterial biomass are therefore attracting considerable interest as environmentally friendly alternatives for contaminant removal. Integrating bacterial biomass with carbon-based materials can lead to the formation of hybrid bio-inorganic composites with enhanced sorption properties due to synergistic interactions between the components [10,11,12].

Recently, particular attention has been paid to yeast biosorbents due to their availability, non-pathogenic nature, and high tolerance to environmental stress. Among them, the red yeast *Phaffia rhodozyma* represents an interesting biological material due to its unique biochemical composition, including the presence of carotenoid pigments and structurally complex cell walls [13,14,15]. These characteristics may influence interactions between biomass and organic pollutants, but the role of these yeast species in composite adsorption systems has not been sufficiently studied.

Despite the growing number of studies on magnetic biochar adsorbents, several important aspects remain poorly understood. In particular, limited information is available regarding the synergistic effects resulting from combining magnetic biochar with microbial biomass and their impact on the adsorption mechanism. Furthermore, many studies have focused primarily on adsorption capacity, failing to provide a detailed analysis of the physicochemical processes regulating contaminant removal or the structural evolution of the material during adsorption and desorption cycles.

The aim of this study was to synthesize and characterize a new magnetic biocomposite based on biochar obtained from aspen wood and *Phaffia rhodozyma* yeast biomass, and to investigate its potential for removing methylene blue from aqueous solutions. Particular attention was paid to analyzing adsorption kinetics, equilibrium behavior, and the influence of operating parameters, as well as to assess the regeneration efficiency and structural changes occurring during repeated adsorption cycles.

We hypothesized that combining magnetically modified biochar with yeast biomass could lead to a multi-step adsorption mechanism involving electrostatic interactions, surface complexation, and π–π interactions associated with the carbon matrix. Furthermore, the presence of the biological component could introduce additional functional groups capable of interacting with dye molecules, potentially increasing the overall adsorption efficiency of the composite material.

## 2. Materials and Methods

### 2.1. Materials

Plant biomass was used as a precursor for the synthesis of the magnetic biocomposite, which was used to obtain the carbonaceous material by pyrolysis. Solutions of iron(II) and iron(III) salts were used as a source of iron ions in the magnetite synthesis process. A 2% sodium alginate solution and a 2% calcium chloride solution were used to immobilize the microorganisms. Methylene blue was used as a model cationic dye in the biosorption studies. Working solutions were prepared in deionized water at concentrations ranging from 75 to 200 mg/dm^3^ without pH adjustment. The following microorganisms were used for microbiological studies: *Phaffia rhodozyma*, *Saccharomyces cerevisiae*, *Bacillus subtilis*, and *Aspergillus niger*. Based on preliminary studies, *Phaffia rhodozyma* was selected as the microorganism with the highest dye removal capacity. All chemicals used in this study were of analytical grade and purchased from Sigma-Aldrich (Merck) (Steinheim, Germany), unless otherwise stated, and were used without further purification. All microorganisms were obtained from the DSMZ—German Collection of Microorganisms and Cell Cultures (Braunschweig, Germany).

### 2.2. Obtaining a Magnetic Biocomposite

#### 2.2.1. Biomass Pyrolysis

The biochar production process was carried out using aspen wood chips as lignocellulosic precursors, selected due to their ready availability as a wood-processing/forestry residue and their suitability as a reproducible woody feedstock for producing stable biochar for low-cost water-treatment applications. In each pyrolysis cycle, a 30 g sample of the raw material was placed in a ceramic cuvette, which was then introduced into the heating zone of a horizontal tube furnace with an internal diameter of 0.1 m (Ströhlein Instruments, Ofen 85, Düsseldorf, Germany). To ensure anaerobic conditions and eliminate thermal oxidation reactions, the system was pre-inerted by purging with argon for 30 min. This procedure allowed for the complete removal of oxygen from the reactor’s working volume. The actual carbonization process was carried out at 550 °C for a retention time of 3 h. After completion of the thermal treatment and cooling of the system to room temperature, the resulting biochar was weighed to determine the process efficiency. The average product mass after a single cycle was 7.5 g, corresponding to a carbonization efficiency of 25%. The entire procedure was repeated four times under identical operating conditions, obtaining a total of 30 g of material for further modification stages and sorption studies.

#### 2.2.2. Obtaining Magnetic Biochar

To impart magnetic properties to the biochar, its surface was modified by in situ precipitation of magnetite (Fe_3_O_4_). The procedure began by weighing 3 g of homogenized biochar into a Teflon vessel, to which 20 cm^3^ of iron precursor solution was added. A mixture of Fe^2+^ and Fe^3+^ salts was used, with concentrations of 0.3 mol/dm^3^ and 0.6 mol/dm^3^, respectively, maintaining a stoichiometric molar ratio of ions of 1:2. The system was incubated for 30 min and then homogenized using an ultrasonic reactor. During sonication, a precipitating agent in the form of a 5.0 mol/dm^3^ NaOH solution was added dropwise to the mixture until a strongly alkaline pH (pH = 11) was achieved. Although aqueous ammonia is commonly used in magnetite co-precipitation protocols, NaOH was selected in this study as the precipitating agent to enable precise and rapid pH adjustment and to avoid the presence of residual ammonium species. The use of NaOH also eliminates NH_3_ volatilization and reduces the risk of undesired interactions with the alginate matrix and biochar surface, thereby improving reproducibility and compatibility with the subsequent immobilization step. The prepared suspension was subjected to microwave-assisted hydrothermal treatment in a pressure reactor (Magnum II, Ertec, Wrocław, Poland). The synthesis process was carried out for 25 min at 180 °C. After the reaction was completed, the resulting magnetic composite was repeatedly washed with deionized water until the leachate was neutralized (pH = 7). The material was dried at 60 °C to a constant mass. After drying, the material was gently ground in an agate mortar to obtain a fine and homogeneous powder suitable for subsequent alginate immobilization. This grinding step was intended to ensure uniform dispersion of the magnetic biochar within the polymer matrix and to avoid large agglomerates that could negatively affect bead formation, mass transfer, and reproducibility of the sorption experiments. The prepared adsorbent was stored in sealed polypropylene containers until sorption tests were performed.

#### 2.2.3. Selection and Preparation of Microbiological Biomass

The initial stage of the study involved selecting the microorganism with the highest bioremediation potential against methylene blue. Four strains were selected for comparative testing: *Bacillus subtilis*, *Aspergillus niger*, *Saccharomyces cerevisiae*, and *Phaffia rhodozyma*. The screening process was carried out in polypropylene vessels by adding 3 cm^3^ of the microorganism suspension to 30 cm^3^ of the dye solution at a concentration of 40 mg/dm^3^. After 1 h of shaking (130 rpm) and filtration, the biosorption efficiency was assessed spectrophotometrically at a wavelength of λ = 664 nm. Based on the highest degree of color reduction, the yeast *Phaffia rhodozyma* was selected for further development.

The selected strain was cultured under sterile conditions in YCU liquid medium. Biological material collected from the YPD solid medium was transferred to conical flasks containing 40 cm^3^ of sterile medium composed of peptone (2 g/dm^3^), yeast extract (2 g/dm^3^), sucrose (16 g/dm^3^), and concentrated mineral salt solution (MgSO_4_, (NH_4_)_2_SO_4_, CaCl_2_, K_2_HPO_4_). The culture was conducted at 25 °C with constant shaking (130 rpm). Biomass growth was monitored visually by increasing the solution turbidity and the appearance of a characteristic orange precipitate, typical of carotenoid synthesis by *P. rhodozyma*. After achieving the required culture density, the biomass was isolated by centrifugation (4000 rpm, 10 min) and prepared for integration with the biochar matrix.

#### 2.2.4. Assessment of Cytotoxicity and Biomass Viability

The study of the viability of *Phaffia rhodozyma* yeast in the presence of methylene blue was conducted using a two-parameter system, taking into account varying dye concentrations (50, 100, 150, 200, and 300 mg/dm^3^) and exposure times (0.5, 1, 1.5, and 2 h). Eppendorf tubes were filled with 1 cm^3^ of the dye solution at the specified concentration and 1 cm^3^ of the yeast suspension, yielding a working volume of 2 cm^3^. Samples were incubated at 35 °C for specified time intervals.

Immediately after incubation, the viable specimens were analyzed using light microscopy (Levenhuk D870T 8M, Levenhuk, Tampa, FL, USA) (400 × magnification). Viability was assessed based on the metabolic capacity of the cells to reduce methylene blue. Viable cells remained colorless due to intracellular reduction of the dye to its colorless leuco form (leukoblue). Dead cells, lacking the ability to transform the dye, were permanently stained blue. Photographic documentation of the visual fields was subjected to quantitative analysis, counting the fraction of live and dead cells, which allowed for the determination of the kinetics of biomass survival under chemical stress conditions.

#### 2.2.5. Synthesis of a Magnetic Biocomposite by Matrix Immobilization Method

The final biocomposite was obtained by immobilizing *Phaffia rhodozyma* yeast biomass on a magnetic biochar matrix using alginate gel trapping. To ensure representative results across all planned sorption tests, the preparation was conducted on a scale ten times larger than the pilot trials, maintaining strictly defined mass proportions of the components. The synthesis procedure involved preparing a homogeneous suspension consisting of 1 g of magnetic biochar, 0.2 g of microbial biomass, and 1.5 g of a 2% sodium alginate solution. After thorough homogenization, the mixture was dosed dropwise into a 2% calcium chloride (CaCl_2_) solution, acting as a cross-linking agent. The applied masses and concentrations of the individual components were selected based on preliminary optimization experiments and previous experience with alginate-based immobilization systems. These conditions were chosen to provide a balanced compromise between sufficient loading of magnetic biochar, preservation of biological activity of the immobilized yeast biomass, and adequate mechanical stability of the alginate matrix. Under these proportions, homogeneous and mechanically stable biocomposite beads were reproducibly obtained while maintaining favorable sorption performance and effective magnetic separability. Contact with calcium ions resulted in immediate gelation of the alginate, leading to the formation of stable, spherical biocomposite structures. The obtained material with a total weight of 30 g was characterized by a uniform distribution of the active phase inside the polymer matrix and was ready for further process tests.

### 2.3. Biosorption Studies

#### 2.3.1. Determination of the Point of Zero Charge (pH_PZC_)

Point of zero charge (PZC) analysis was performed to determine the amphoteric surface properties of the resulting biocomposite and to identify the pH at which the total surface charge of the material is zero. The procedure began with the preparation of a neutral electrolyte solution (NaCl, 0.05 mol/dm^3^) and titrant solutions (HCl and NaOH, 0.1 mol/dm^3^).

In the first phase, a series of stock solutions were prepared with determined initial pH values (pH_0_) ranging from 2 to 12 (in 2-unit increments). pH correction was performed using microdosing of HCl or NaOH, while monitoring parameters with a pH meter (MARTINI Mi 150). Then, 20 cm^3^ of the solutions with the desired pH and 0.6 g of the biocomposite samples were added to sealed polypropylene containers. The systems were subjected to continuous shaking (130 rpm) at room temperature for 24 h, which allowed for adsorption equilibrium to be achieved and charge stabilization at the solid–liquid interface. After the incubation period, the final pH (pH_e_) of the solutions was measured. The pH_PZC_ value was determined graphically by analyzing the pH change (ΔpH = pH_0_ − pH_e_) as a function of initial pH. The intersection of the obtained curve with the *x*-axis (ΔpH = 0) was defined as the point of zero charge for the tested material [16].

#### 2.3.2. Effect of Biocomposite Dose

Determining the optimal adsorbent dose is a key step in designing sorption processes, determining their unit efficiency, overall contaminant removal efficiency, and economic aspects. To determine the effect of solid phase mass on the kinetics and equilibrium of the process, a series of samples was prepared containing increasing biocomposite weights: 0.2, 0.4, 0.6, 0.8, and 1.0 g. Each polypropylene reaction vessel was charged with 20 cm^3^ of methylene blue solution with an initial concentration of C_0_ = 100 mg/dm^3^. The process was conducted under dynamic conditions (laboratory shaker, 130 rpm) in two time variants: 1 h (preliminary kinetics control) and 24 h (equilibrium achievement). After incubation, the residual dye concentration was determined by UV–Vis spectrophotometry at an analytical wavelength of λ = 664 nm. Based on the obtained absorbance data, key process parameters were calculated: dye removal efficiency (RE [%]) and sorption capacity (q_e_ [mg/g]), according to the equations provided in Supplementary Table S1. Analysis of these parameters allowed for the selection of the optimal sorbent dose, ensuring the most favorable ratio of solution purification to the mass of used material.

#### 2.3.3. Adsorption Kinetics Studies

Kinetic studies were conducted to thoroughly assess the effect of phase contact time on the dynamics of the sorption process and to identify the mechanism controlling the rate of binding of methylene blue molecules by the structure of the produced biocomposite. The experimental procedure began by weighing a constant dose of 0.6 g of adsorbent into a series of ten polypropylene containers, each of which was then filled with 20 cm^3^ of dye solution with an initial concentration of C_0_ = 100 mg/dm^3^. The samples were continuously mixed under dynamic conditions on a laboratory shaker at 130 rpm, with the process interrupted at precisely defined time intervals: 0.083, 0.25, 0.5, 1, 2, 3, 4, 5, and 24 h. After each incubation step, the residual methylene blue concentration was determined spectrophotometrically at an analytical wavelength of λ = 664 nm. The obtained experimental data were subjected to regression analysis using four classical kinetic models, including the pseudo-first-order (PFO) and pseudo-second-order (PSO) models, which allowed for verification of the potential dominance of the chemisorption mechanism. Additionally, the Elovich model, dedicated to highly energetically heterogeneous surfaces, and the Weber–Morris intraparticle diffusion model were used to identify the rate-limiting steps of the process, such as external diffusion or transport within the material’s pores (Table S1).

#### 2.3.4. Adsorption Isotherm Studies

Adsorption equilibrium studies were conducted to determine the maximum sorption capacity of the produced biocomposite and to determine the nature of interactions occurring at the solid–liquid interface. The procedure began with the preparation of a series of methylene blue solutions with varying initial concentrations: 75, 100, 125, 150, 175, and 200 mg/dm^3^. A constant dose of 0.6 g of the adsorbent was weighed into six polypropylene containers, and then 20 cm^3^ of the solution at the specified concentration was added to each container. The sorption process was conducted isothermally at 25 °C with constant shaking, using two time variants: 1 h and 24 h, which allowed for the detection of differences between the transition state and full thermodynamic equilibrium. After incubation, the residual dye concentration was determined spectrophotometrically at an analytical wavelength of λ = 664 nm. The obtained experimental data were analyzed using four classical adsorption isotherm models, which enabled a mathematical description of the process. The Langmuir model was used to verify the formation of a monolayer on active sites, and the Freundlich model, dedicated to multilayer adsorption on heterogeneous surfaces. Additionally, the Temkin model, which takes into account the interactions between the adsorbate and the adsorbent, and the Dubinin–Radushkevich model were used to estimate the free energy of the process and distinguish the mechanisms of physisorption from chemisorption (Table S1).

#### 2.3.5. Effect of Temperature

The effect of temperature on the sorption process was studied to assess the thermodynamics of the system and determine the operational stability of the biocomposite, particularly in the context of the presence of a biological fraction in the form of *Phaffia rhodozyma* yeast cells. Because the metabolic activity of microorganisms and the stability of their cell walls are strongly dependent on thermal conditions, experiments were conducted over a wide temperature range: 20, 25, 30, and 35 °C. A series of polypropylene containers were each loaded with 20 cm^3^ of methylene blue solution with an initial concentration of C_0_ = 100 mg/dm^3^. Before the actual sorption process began, the dye solutions were thermally conditioned in a water bath until the desired parameters were achieved. After temperature stabilization, a 0.6 g sample of biocomposite was added to each system. The adsorption process was conducted for 3 h while maintaining constant thermal control of the liquid, eliminating errors resulting from ambient temperature fluctuations. The residual concentration of methylene blue was determined by UV–Vis spectrophotometry at a wavelength of λ = 664 nm. The obtained experimental results were used to determine the sorption capacity (q_e_ [mg/g]) and to analyze the correlation between the thermal energy of the system and the process efficiency.

#### 2.3.6. Regeneration Research

Analyzing the regeneration and reuse processes of an adsorbent is a key element in assessing its applicability in industrial systems, combining economic aspects with the sustainability paradigm. To identify the most effective desorption agent, four eluent variants were used in the study: hydrochloric acid (HCl, 0.1 mol/dm^3^), nitric acid (HNO_3_, 0.1 mol/dm^3^), 48% ethanol solution (C_2_H_5_OH), and deionized water. The procedure began with the preparation of a saturated biocomposite through 3-h sorption of methylene blue at an initial concentration of C_0_ = 100 mg/dm^3^. After the sorption process, the material was pre-washed with deionized water to remove unbound dye molecules and then dried to constant weight. A series of polypropylene containers were weighed with 0.6 g of the prepared sorbent and each was filled with 20 cm^3^ of the selected regenerating agents. The systems were subjected to vigorous shaking (130 rpm) for 30 min to force the desorption of methylene blue from the active sites of the biocomposite. Utilizing the magnetic properties of the material, the adsorbent was separated from the liquid phase using an external magnetic field, and the desorption solution was subjected to spectrophotometric analysis at a wavelength of λ = 664 nm. The regenerated biocomposite was washed until a neutral pH was achieved, dried, and sent to another 3-h sorption cycle. This procedure was repeated several times to determine the structural stability of the material and to calculate the percentage desorption efficiency in subsequent operating cycles, until significant physicochemical degradation of the sorbent was observed.

### 2.4. Material Characterization

Advanced analytical and instrumental methods were used to comprehensively assess the structure, morphology, and magnetic-sorption properties of the obtained materials. Characterization of the lignocellulosic precursors began with thermal analysis (TA) of raw aspen wood in an argon atmosphere. Thermal analysis was carried out with an EXSTAR SII TG/DTA 7300 analyzer (Dallas, TX, USA) under a continuous argon flow at a heating rate of 20 °C/min in the temperature range of 30–1000 °C. This study allowed for the precise determination of the thermal decomposition profile of the biomass, identification of the ranges of moisture loss, and the degradation of hemicellulose, cellulose, and lignin, which formed the basis for optimizing the pyrolysis process conditions. The surface morphology and degree of porosity development of the resulting biocomposite were analyzed using scanning electron microscopy (SEM) (Hitachi TM-3000, Tokyo, Japan). This technique enabled the visualization of textural changes and the assessment of the structural stability after adsorption and repeated regeneration processes. In parallel, infrared spectroscopic studies (FTIR) were conducted to identify key functional groups present in the carbon matrix and in the cellular structures of the yeast *P. rhodozyma* (Thermo Scientific—Nicolet iS5 with the ATR iD7 attachment) (Waltham, MA, USA). Spectral analysis allowed for the tracking of vibrations characteristic of bonds actively involved in the chemical bonding of dye molecules. The phase composition and degree of crystallinity, with particular emphasis on the presence of magnetite (Fe_3_O_4_), were verified using X-ray diffraction (XRD). X-ray diffraction analysis was conducted with a Rigaku SmartLab SE system equipped with a Hypix 400 semiconductor detector (Rigaku, Tokyo, Japan). Magnetic parameters, which determine the efficiency of adsorbent separation from the aqueous phase, were determined using a vibrating magnetometer (VSM) (VSM, Lakeshore 7407, Lake Shore Cryotronics, Westerville, OH, USA). Quantitative determination of methylene blue concentration was performed using UV–Vis spectrophotometry at an analytical wavelength of λ = 664 nm (Rayleigh UV-1800, Chelmsford, UK). These measurements, based on the determined regression curve, formed the basis for calculating the process efficiency and determining the kinetic and equilibrium sorption parameters.

## 3. Results and Discussion

### 3.1. Selection and Survival of Microorganisms in the Biocomposite

**Figure 1:**
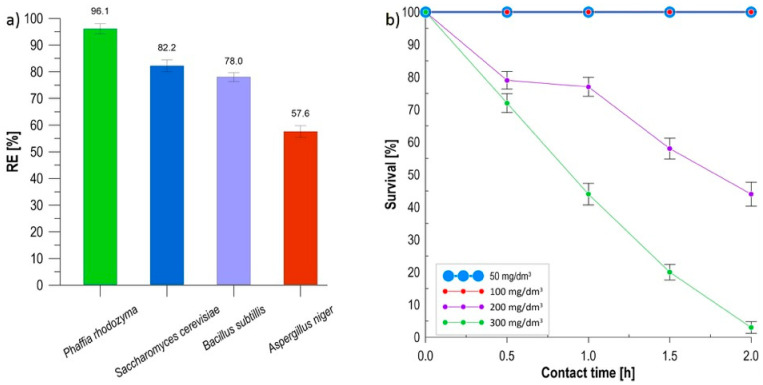
(**a**) MB removal ability of selected microorganism strains, (**b**) survival of *P. rhodozyma* against MB.

The initial stage of the study focused on selecting the strain with the highest bioremediation potential and assessing its individual resistance to the cytotoxic effects of methylene blue. Selecting appropriate microorganisms is crucial for the subsequent formulation of a stable biocomposite, as the high metabolic activity of the inoculum directly determines the efficiency of contaminant removal in hybrid systems [17]. The dye removal efficiency (RE [%]) for the four selected strains (Figure 1a) was calculated using a mass balance equation. The highest color reduction efficiency, 96.1%, was recorded for the yeast *Phaffia rhodozyma*, which can be attributed to the complex composition of its cell wall, rich in glucans and specific functional groups [18]. This result was significantly higher compared to the other microorganisms: *Saccharomyces cerevisiae* (82.2%), *Bacillus subtilis* (78.0%), and *Aspergillus niger* (57.6%). Due to its dominant sorption properties, the *P. rhodozyma* strain was selected as the active biological component for further research. Another key aspect was the assessment of the survival of selected yeasts in direct contact with the dye (Figure 1b). Microscopic analysis of the in vivo preparations showed that in solutions with lower concentrations (50 and 100 mg/dm^3^), the yeasts remained fully viable (100% viable cells) throughout the entire test period (2 h). Under these conditions, the microorganisms effectively neutralized the dye, as evidenced by a lack of cytosolic staining [19]. A significant decrease in survival was observed at higher concentrations of methylene blue. At a dose of 300 mg/dm^3^, the fraction of dead cells increased from 28% after 0.5 h to as much as 97% after 2 h of exposure. This mechanism was directly correlated with the loss of metabolic capacity for intracellular dye reduction, resulting in a permanent blue discoloration of dead cells. The observed increase in the percentage of dead cells by 69 percentage points at the highest concentration confirms the significant impact of chemical stress on the biological condition of the inoculum [20]. The study showed that despite high sorption efficiency, dye concentrations exceeding 200 mg/dm^3^ constitute a barrier to maintaining the full metabolic activity of free *P. rhodozyma* yeast cells. The data obtained provide a reference point for further research on microorganism immobilization, suggesting the need for a supporting matrix that could serve a protective function and increase the biological resistance of the system under conditions of high contaminant load.

### 3.2. Selection of Component Parameters and Functional Evaluation of the Biocomposite

The process of designing a multicomponent sorbent required a precise balance between the magnetic carrier, polymer matrix, and biological component. A key research challenge was identifying the equilibrium point at which the biocomposite maintains high magnetic controllability while simultaneously maximizing the number of available active sites. Process efficiency was monitored by determining the sorption capacity q [mg/g] and the percentage color reduction RE [%] [21].

#### 3.2.1. Influence of Biochar Mass Fraction on Matrix Properties

In the first stage, the effect of magnetic biochar content (in the range of 0.1–0.4 g) was analyzed while maintaining a constant amount of sodium alginate (1.0 g). According to the results summarized in Table S2, variant 1 demonstrated the highest sorption efficiency, with RE = 28.7% and q = 2.64 mg/g. A clear negative correlation was observed between the mass of the carbon phase and the sorption capacity of the composite. Increasing the biochar content to 0.4 g resulted in a reduction in yield by over 7 percentage points. This phenomenon can be explained by steric blocking [22]. Despite its porosity, biochar in this particular system primarily served as a carrier for magnetic particles. Its excess in the alginate structure likely caused physical obscuration of the polymer’s functional groups (primarily carboxyl and hydroxyl), which constitute the main binding sites for the cationic dye. Variant 1 was considered optimal because despite the lowest biochar content, the material exhibited sufficient magnetic properties to allow for effective separation from the solution.

#### 3.2.2. The Role of *Phaffia rhodozyma* Biomass and Inoculum Optimization

Studies on the effect of inoculum mass fraction (range 0.1–0.4 g) confirmed the synergistic effect of the biological and synthetic components (Tables S3 and S4). Analysis conducted over two time intervals revealed that variant 2, containing 0.2 g of microorganisms, had the most favorable parameters. After 24 h of processing, this material achieved an efficiency of RE = 58.2% and a sorption capacity of q = 1.17 mg/g. It was found that both a deficiency (variant 1) and an excess of biomass (variants 3 and 4) led to a decrease in process parameters. The decrease in efficiency at doses above 0.2 g suggests the occurrence of coagulation or excessive cell crowding within the alginate matrix. An excess of microorganisms can saturate easily accessible active sites and impede the diffusional transport of the dye to the deeper layers of the biocomposite [23]. At the same time, the increased efficiency of variant 2 compared to variant 1 (37.6% vs. 40.4% after 1 h) confirms that the yeast actively supports the color reduction process.

#### 3.2.3. The Importance of Cell Density and Verification of Biological Contribution

The final stage of optimization concerned the inoculum preparation method. The results presented in Tables S5 and S6 clearly indicate the advantage of using centrifuged biomass (variant no. 2), which allowed for the achievement of the highest values throughout the entire research cycle: RE = 62.8% and q = 2.96 mg/g after 24 h of the process. The use of centrifuged *P. rhodozyma* cells allows for a significantly higher density of active biosorption sites per unit volume of the biocomposite. Key evidence for the active role of yeast is provided by a comparison with the reference sample (variant no. 3), containing deionized water instead of microorganisms. The addition of centrifuged biomass increased the sorption efficiency by over 13 percentage points (from 49.7% to 62.8%). This indicates that microorganisms do not merely act as a passive filler but constitute an active sorption phase, which, thanks to specific functional groups in their cell walls, significantly enhances the purification properties of the entire system [24].

### 3.3. Analysis of the pH_PZC_ Point

**Figure 2:**
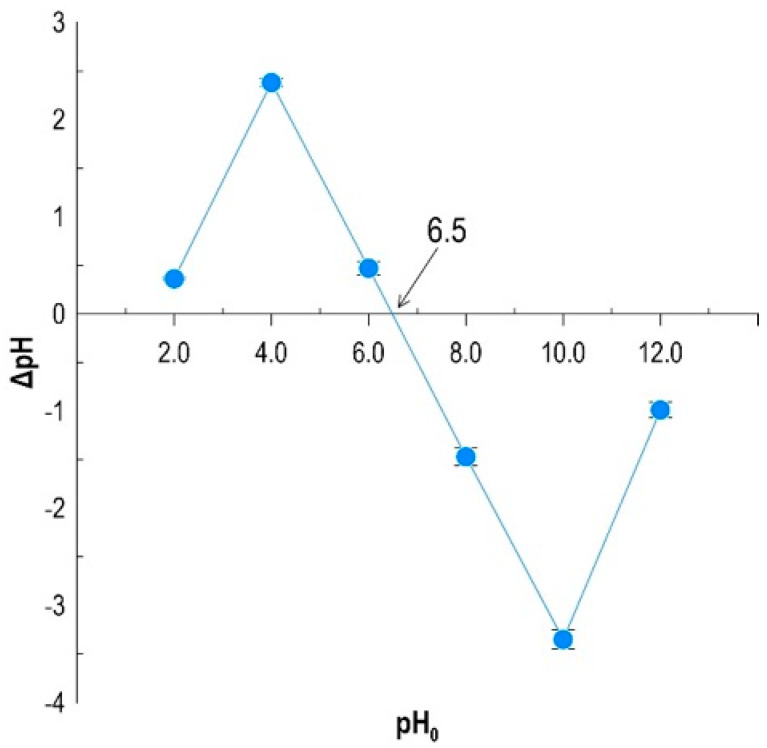
pH_ZPC_ of the biocomposite.

Determining the point of zero charge (pH_PZC_) allowed for a precise determination of the acid–base nature of the biocomposite surface and an understanding of the mechanism of its interactions with cationic dyes. Based on the results of pH measurements of solutions after 24 h of contact with the adsorbent, the dependence of the pH difference (ΔpH) on the initial pH_0_ value was plotted (Figure 2). Graphical analysis identified the intersection of the obtained curve with the *x*-axis (ΔpH = 0), which for the tested material was 6.5. This value is a key operational parameter, determining the efficiency of sorption processes depending on the environmental pH. In the pH range > 6.5, the biocomposite surface exhibits a total negative charge. This results from the deprotonation of functional groups present in the alginate matrix and on the surfaces of biochar and yeast cell walls. Under these conditions, strong electrostatic attractive forces develop between the negatively charged sorbent and the cationic methylene blue molecules, which directly translates into high dye removal efficiency [25]. However, at pH < 6.5, the material surface becomes protonated, acquiring a positive charge. This leads to electrostatic repulsion with the positively charged methylene blue groups, which can result in reduced sorption capacity in a strongly acidic environment. The identified pH_PZC_ value of 6.5 indicates that the developed biocomposite possesses amphoteric properties with a slightly acidic charge equilibrium point. This suggests that optimal conditions for methylene blue sorption occur in solutions with a pH close to neutral (pH ≈ 7), where the sorbent surface is already negatively charged, maximizing chemical affinity for the contaminant [26]. These data correlate with the results of studies on the influence of the biocomposite composition, confirming that the electrostatic mechanism plays an important role in addition to the biosorption carried out by immobilized *Phaffia rhodozyma* cells.

### 3.4. The Influence of the Biocomposite Dose on the Dye Removal Efficiency

**Figure 3:**
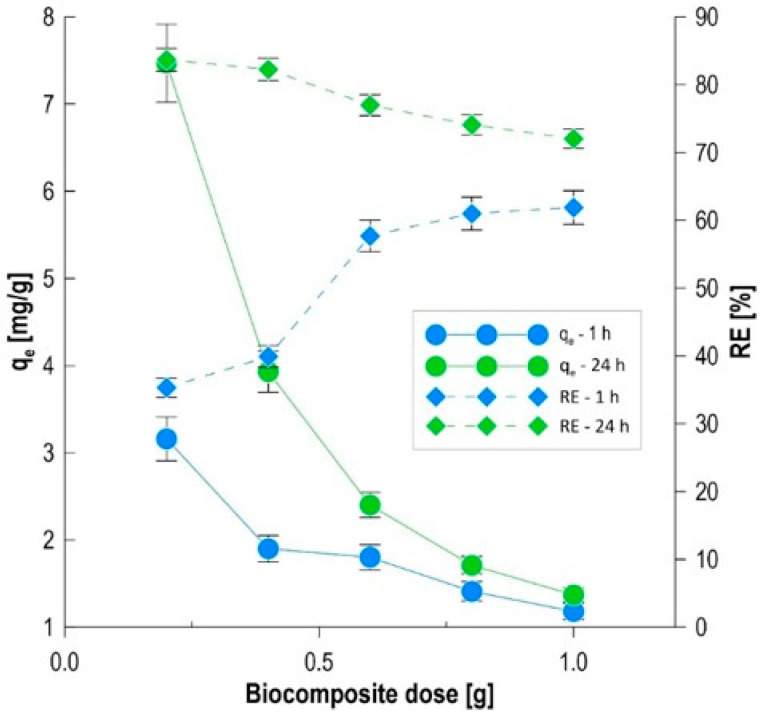
The effect of biocomposite dose on q_e_ and RE.

Analysis of the obtained experimental data allowed us to determine the correlation between the mass of the sorbent used and the efficiency of methylene blue removal from the aqueous phase. The results, summarized in Figure 3, illustrate the change in the RE and q_e_ parameters over time, enabling the assessment of the material’s efficiency both in the dynamic growth phase and in the stabilized state. The initial phase of the process (1 h) demonstrated a relationship between the dose increase and the degree of color reduction. Increasing the sample size from 0.2 g to 1.0 g resulted in a nearly twofold increase in efficiency, from 35.3% to 61.9%. This phenomenon is directly correlated with the increase in the total number of available active sites and functional groups (derived from alginate and biomass), which are capable of binding dye cations on the biocomposite surface in a short contact time [27]. After 24 h of the process, a significant leveling of the solution purification degree was observed for all tested variants, with these values ranging from 72.04% to 83.68%. The highest RE index after equilibrium was achieved for the lowest dose (0.2 g), suggesting that the biosorption potential of the immobilized microorganisms and the carbon matrix is maximized over time. Analysis of the sorption capacity revealed a trend inversely proportional to the adsorbent mass. At equilibrium, the q_e_ value decreased from 7.47 mg/g for the 0.2 g dose to 1.37 mg/g for the 1.0 g dose. This pattern of changes, typical of heterogeneous systems, results from several overlapping factors, including surface unsaturation, where at high sorbent doses there is a large excess of active sites relative to the number of dye molecules in the solution, which prevents achieving maximum material saturation, and the shielding and aggregation effects, which cause a higher concentration of biocomposite beads in a limited sorbate volume to promote their mutual contact, which can lead to a reduction in the effective contact surface and hinder the internal diffusion of adsorbates [28,29]. A comparison of the kinetic and final results indicates a 0.4 g dose as optimal for the designed system. This variant is characterized by high process stability, ensuring a color reduction of 82.26% while maintaining a favorable sorption capacity (3.93 mg/g). The selection of this sample represents a compromise between the rate of contaminant removal visible in the first hour of the process and the economical use of the sorbent over the 24-h cycle.

### 3.5. Kinetic Studies of the Biosorption Process

**Figure 4:**
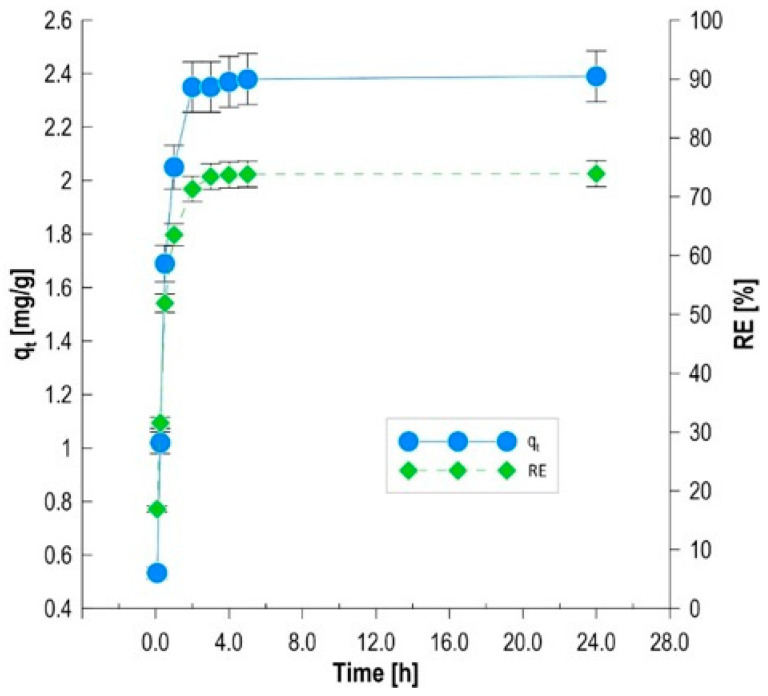
MB removal by the biocomposite as a function of time.

Time-course analysis of methylene blue removal by a biocomposite enriched with *Phaffia rhodozyma* yeast cells allowed for the assessment of the dynamics of solid–liquid interactions and the identification of mechanisms controlling the sorption rate (Figure 4). Studies limited to a 24-h time horizon allowed for the precise determination of kinetic parameters during the phase of most intense adsorbate binding and toward thermodynamic equilibrium. The biosorption process during the studied time interval was characterized by high initial intensity, which is typical for materials with a developed and heterogeneous surface [30]. Within the first hour of contact, a rapid increase in sorption capacity was observed, from 0.53 mg/g (after 5 min) to 2.05 mg/g. Dye removal efficiency (RE) increased from 16.9% to 63.5% during this time. This high dynamic results from the immediate occupation of readily accessible active sites on the external surface of the biocomposite by dye cations. After the first hour, the binding rate gradually slowed, resulting from the gradual saturation of functional groups and increased diffusion resistance within the alginate–carbon matrix [31]. After 24 h, the system reached a stable sorption capacity of 2.39 mg/g, which translated into a total color reduction of 73.9%. The negligible efficiency gains after this time suggest that 24 h can be considered an optimal practical contact time for the designed sorbent. It should be noted that the apparent equilibrium reached after approximately 180 min corresponds to a quasi-equilibrium state, where the adsorption rate becomes very low. The additional increase in sorption capacity observed after 24 h reflects slower processes, including intraparticle diffusion within the alginate–biochar matrix and biosorption associated with the immobilized yeast biomass, which continue beyond the initial kinetic stabilization.

**Figure 5:**
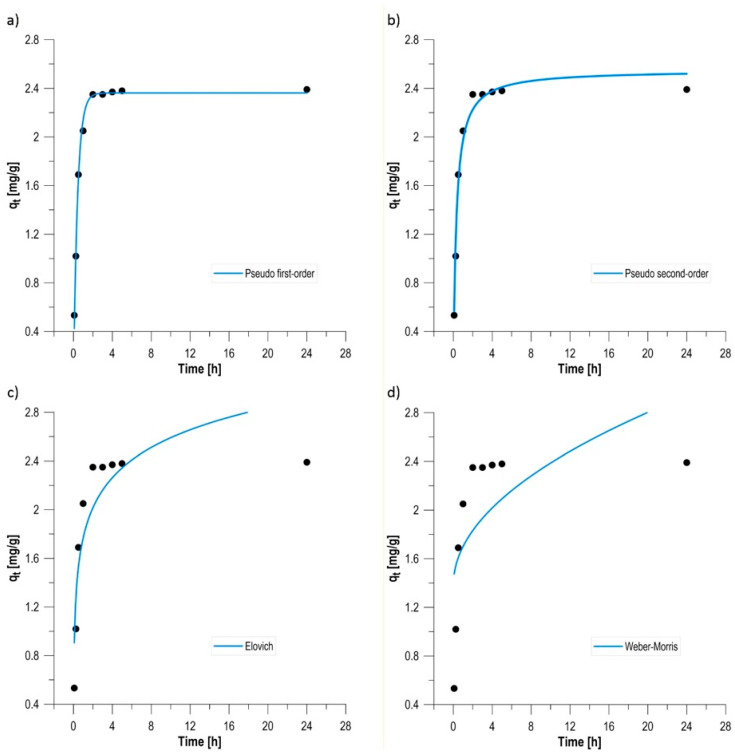
Graphs illustrating the course of the process kinetics: (**a**) pseudo first-order, (**b**) pseudo-second order, (**c**) Elovich, and (**d**) Weber–Morris.

To verify the nature of the interactions between the biocomposite and the dye and to identify the rate-limiting step in the process, the experimental data were analyzed using four nonlinear kinetic models (Figure 5). This method avoids statistical distortions, resulting in a more reliable physicochemical interpretation of the system [32]. A key criterion for assessing the binding mechanism is the comparison of the fit of the first-order (PFO) and second-order (PSO) models. The quality of model fitting was evaluated using the coefficient of determination (R^2^) and the average relative error (ARE), which quantifies the deviation between experimental and model-predicted sorption capacities. The definition and calculation procedure for ARE and R^2^ are provided in Table S1. Lower ARE values indicate a better agreement between the model and experimental data. Based on the obtained parameters (Table 1), it was found that the pseudo-first-order (PFO) model demonstrated the best fit to the experimental data, achieving a coefficient of determination of R^2^ = 0.9966. The very low ARE error (3.86%) and the calculated sorption capacity of q_1_ = 2.36 mg/g indicate that this model best describes the system dynamics in the studied range. This suggests that the process rate is proportional to the number of free active sites, which is often associated with the dominance of diffusive physicochemical processes in the initial phase of sorption. The pseudo-second-order (PSO) model, despite its high R^2^ = 0.9906, exhibits a higher fitting error (ARE = 4.78%) compared to the PFO model. The determined rate constant k_2_ was 1.34 g/(mg·min), and the theoretical sorption capacity q_2_ = 2.55 mg/g. Although the fit is satisfactory, the lower precision compared to the PFO model may suggest that chemisorption is not the only factor controlling the kinetics [33]. Additional information on the sorbent structure and mass transfer resistance was obtained using the Elovich and Weber–Morris models. In the Elovich model, the obtained R^2^ = 0.8979 and ARE = 18.75% error indicate a significant role of the biocomposite surface heterogeneity. The α parameter (initial sorption rate) of 49.72 mg/(g·min) confirms the high reactivity of the material in contact with methylene blue. This model confirms that the sorbent surface, consisting of alginate, biochar, and yeast biomass, has differentiated active site energies [34]. The intrapore diffusion (Weber–Morris) model demonstrated the poorest fit (R^2^ = 0.6421) with the highest error, ARE = 37.71%. The parameter I = 1.38 mg/g (boundary layer thickness) was nonzero, clearly demonstrating that intrapore diffusion is not the only process-limiting step. The overall dye removal rate is influenced by a combination of diffusion in the boundary layer (film diffusion) and surface interactions at the interface [35]. The use of nonlinear kinetic models allowed for a precise ranking of the mechanisms controlling the process in the following order: pseudo-first-order > pseudo-second-order > Elovich > Weber–Morris. The dominance of the PFO model with the support of the Elovich model parameters proves that the process of methylene blue removal on the developed biocomposite is a complex process in which the availability of active centers on the heterogeneous surface of the material plays a key role.

### 3.6. Equilibrium Studies of the Sorption Process

**Figure 6:**
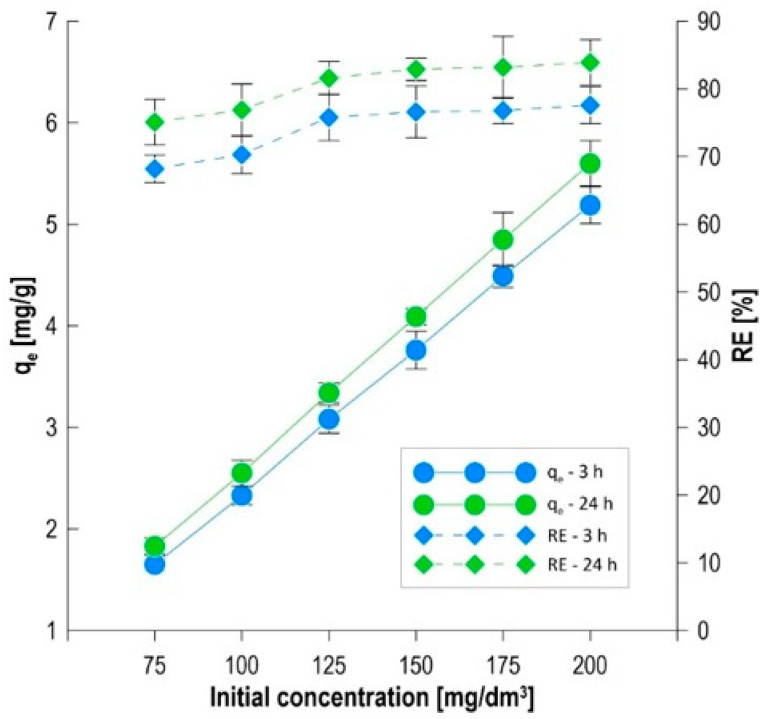
MB removal by the biocomposite over 3 h and 24 h at variable C_0_.

Analyzing the effect of the initial adsorbate concentration on process efficiency is a key element in assessing the application potential of a biocomposite, allowing for the determination of its limiting sorption capacity and understanding the mass transfer mechanisms. The studies were conducted over a wide range of methylene blue concentrations (75–200 mg/dm^3^), allowing for the observation of the system’s behavior under both a deficiency and a significant excess of dye molecules relative to the available active sites. The initial equilibrium phase, analyzed after 3 h of the process, showed a dynamic increase in the amount of adsorbed dye with an increase in its concentration in the solution (Figure 6). At a concentration of 75 mg/dm^3^, the sorption capacity was 1.65 mg/g, corresponding to a color reduction (RE) of 68.2%. Increasing the concentration to 200 mg/dm^3^ more than tripled the amount of bound contaminant, reaching q_e_ = 5.19 mg/g. This phenomenon is directly correlated with the increasing driving force of the process, which is the concentration gradient between the liquid phase and the sorbent surface. A higher initial concentration intensifies the diffusion of methylene blue molecules through the solution boundary layer, enabling the faster occupation of active sites located in the pores of the alginate–carbon matrix [23]. Extending the contact time to 24 h allowed the system to fully stabilize and achieve thermodynamic equilibrium (Figure 6). For the lowest concentration (75 mg/dm^3^), an increase in qe to 1.83 mg/g was observed, with a simultaneous increase in removal efficiency to 75.1%. In the variant with the highest loading (200 mg/dm^3^), the sorption capacity reached 5.60 mg/g, providing an 83.9% color reduction. Comparison of the results after 3 h and 24 h indicates the significant role of the yeast *Phaffia rhodozyma* as the biological component. While physical adsorption on biochar occurs rapidly in the initial hours, biosorption and accumulation processes within the biomass require a longer contact time, resulting in a significant increase in efficiency in the second phase of the experiment [36]. It is worth noting that the highest concentration (200 mg/dm^3^) recorded the highest degree of color reduction throughout the entire 24-h cycle (83.9%). This demonstrates the biocomposite’s exceptionally high chemical affinity for methylene blue and that even with significant contaminant loading, the material does not become completely saturated. This suggests the presence of an extensive porous structure and a multi-level system of active sites, where the carboxyl groups of alginate and the aromatic structures of biochar interact with the polymers of the microorganisms’ cell walls [20,37].

**Figure 7:**
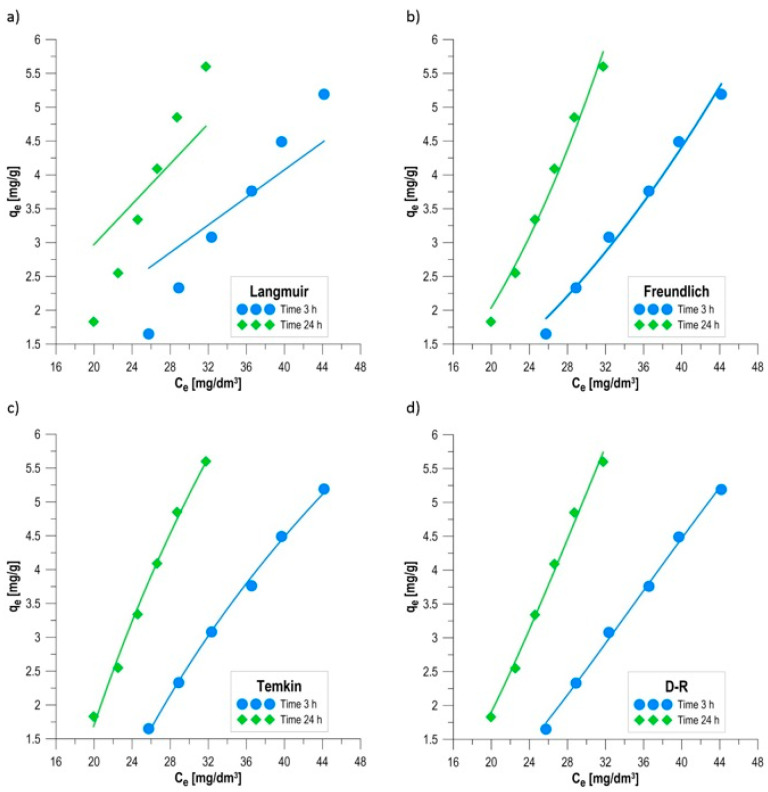
Isotherm models obtained in 3 h and 24 h sorption: (**a**) Langmuir, (**b**) Freundlich, (**c**) Temkin, and (**d**) Dubinin–Radushkevich.

The use of nonlinear regression analysis allowed for precise fitting of isothermal models to experimental data, enabling the identification of mechanisms controlling equilibrium in the methylene blue–biocomposite system. The application of the mean error criterion (ARE) and the coefficient of determination (R^2^) allowed for an objective ranking of the models in terms of their descriptive ability (Table 2) [38]. Figure 7 graphically illustrates the results obtained from fitting the equilibrium models. The modeling results indicate that the methylene blue sorption process is most accurately described by models that account for the energetic heterogeneity of the sorbent surface. The Temkin model demonstrated the highest precision of fit, as confirmed by the lowest relative error values: ARE = 1.59% (R^2^ = 0.9984) after 3 h and ARE = 7.45% (R^2^ = 0.9967) after 24 h. The B constant, representing the heat of adsorption, increased with time from 6.567 kJ/mol to 8.406 kJ/mol. These values are within the range typical of exothermic physicochemical interactions [39]. The increase in binding energy with time suggests that the dye molecules penetrate the biocomposite structure, reaching active sites with higher chemical affinity, located within the matrix or in the cell walls of *Phaffia rhodozyma*. The Dubinin–Radushkevich (D–R) model achieved a very similar degree of fit to the Temkin model (ARE = 2.02% and 7.96%). The key parameter here is the theoretical sorption capacity q_d_, which reached 39.308 mg/g after 24 h. The clear difference between q_d_ and the experimental results indicates that the biocomposite has a significant reserve of active sites, the full saturation of which would require the use of much higher initial dye concentrations [40]. The Freundlich model, despite a high coefficient of determination (R^2^ > 0.99), was characterized by higher relative error values (ARE = 4.10 and 8.39%) than the Temkin and D–R models. The 1/n parameter assumed values in the range of 1.937 and 2.267, which significantly deviated from the values typically found in the literature, which are below unity. Exceeding the threshold of 1/n = 1 indicates cooperative adsorption [41]. This suggests a mechanism in which already adsorbed methylene blue molecules, through intermolecular interactions (e.g., π–π interactions between the aromatic rings of MB), facilitate the binding of subsequent dye cations to the heterogeneous biocomposite surface [42]. The Langmuir model demonstrated the lowest usefulness in describing the studied system, as confirmed by the highest ARE error values (17.14% and 25.31%) and the lowest coefficients of determination (R^2^). The obtained results indicate that this model is inadequate and does not correctly describe the adsorption process in the analyzed system. The inappropriateness of the Langmuir model results from its basic assumptions, i.e., energetic homogeneity of the adsorbent surface, equivalence of all active sites, and the absence of intermolecular interactions. These assumptions were not met in the case of the studied biocomposite with a complex structure (alginate/biochar/yeast), characterized by high chemical and structural heterogeneity. Additionally, the obtained physically unrealistic values of the Langmuir model parameters indicate an incorrect fit of the model to the experimental data, confirming its limited interpretative usefulness in this case. Therefore, it can be concluded that the methylene blue removal process does not follow the mechanism of monolayer adsorption on a homogeneous surface, but rather occurs on an energetically heterogeneous surface, likely involving multiple types of active sites and with the possibility of forming multilayer structures [24]. The lowest ARE error values for the Temkin and D–R models confirm the dominant contribution of exothermic electrostatic and physical interactions, supported by the synergistic action of the porous structure of the biochar and the functional groups of the immobilized biomass. Alternative statistical indicators, such as the reduced chi-square (χ^2^ᵣ), are commonly used in adsorption studies; however, for nonlinear regression based on sorption capacity data, ARE combined with R^2^ provides a robust and sensitive criterion for comparing model performance, leading to the same qualitative ranking of the fitted kinetic models.

A comparison with selected literature reports shows that MB uptake values span a wide range, strongly depending on material architecture and on how adsorption performance is reported. For example, magnetic biochar prepared from mushroom substrate was reported with a methylene blue adsorption value of 2297.04 µg/g (≈2.30 mg/g), which is comparable in magnitude to the qe obtained in the present work under the applied dose and concentration window [43]. Activated carbon–sodium alginate (AC–SA) beads derived from date pits reached a maximum methylene blue adsorption capacity of 23.529 mg/g, a value consistent with the range reported for other alginate-based biocomposite beads [44]. In addition, biopolymer-based adsorbents reported in the literature show capacities such as 36.25 mg/g for lignin–chitosan pellets, further emphasizing that direct capacity comparisons should account for differences in composition (carbon vs. biopolymer), form (powder vs. beads), and experimental conditions [45].

### 3.7. Effect of Temperature on MB Removal

**Figure 8:**
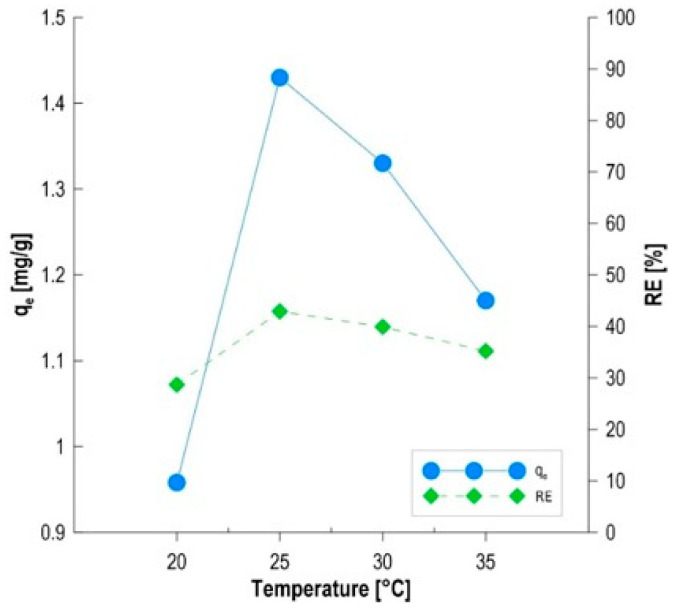
The effect of temperature on MB sorption.

Analysis of the effect of temperature on the efficiency of methylene blue removal by the biocomposite is a key complement to the earlier stages of the research, confirming the hybrid mechanism of the process, in which the synergy of biotic and abiotic components plays a key role (Figure 8). The obtained results demonstrate a close correlation with the previous selection of *Phaffia rhodozyma* biomass and studies of its survival under chemical stress conditions. The observed temperature optimum at 25 °C (RE = 42.9%, q_e_ = 1.43 mg/g) directly coincides with the conditions of the highest metabolic activity of the *P. rhodozyma* yeast, identified in the earlier stage as the most effective inoculum (96.1% reduction in free color). The decrease in process efficiency both below and above this temperature proves that the main mechanism limiting the efficiency of the entire biocomposite is the kinetics of biosorption and the physiological state of the microorganisms, and not solely physical adsorption on the biochar [46]. The temperature dependence of qe is nonlinear, suggesting that the lowest observed efficiency at 20 °C (RE = 28.7%) results from the limited diffusion of methylene blue cations through the solution boundary layers and slower mass transfer into the alginate matrix. In this range, a 5 °C temperature increase provides the necessary kinetic energy to overcome diffusion barriers, resulting in a significant increase in sorption capacity by nearly 50% [47]. The optimum at 25 °C is characteristic of mesophilic biosorption systems. At this temperature, the polymer structures of yeast cell walls (mannans and glucans) exhibit optimal spatial conformation, which maximizes the exposure of negatively charged functional groups previously identified in the pH_PZC_ analysis [48]. The observed decrease in efficiency above 30 °C, where at 35 °C RE = 35.2% and q_e_ = 1.17 mg/g were obtained, suggests two overlapping phenomena. First, the process may be exothermic, with an increase in the system’s thermal energy promoting physical desorption of MB molecules from the biochar surface [49]. Second, a temperature of 35 °C may induce changes in the permeability of yeast cell membranes or lead to their premature autolysis, drastically reducing the number of active biosorption sites [50]. This is consistent with the results of viability tests, which demonstrated the high sensitivity of *P. rhodozyma* to stress factors. The thermal parameters of the process confirm that the developed biocomposite is a highly responsive system, in which the biological phase determines the optimal operating conditions. The demonstrated consistency between the temperature optimum and yeast physiology allows us to conclude that the methylene blue removal mechanism is based on controlled biosorption, supplemented by physical adsorption in the biochar pores. These data, combined with nonlinear isothermal models (Temkin and D–R), create a complete profile of the sorption characteristics of the tested material, indicating 25 °C as the critical temperature for maintaining the stability and efficiency of the system.

### 3.8. Research on the Regenerative Capacity and Desorption Efficiency of the Biocomposite

**Figure 9:**
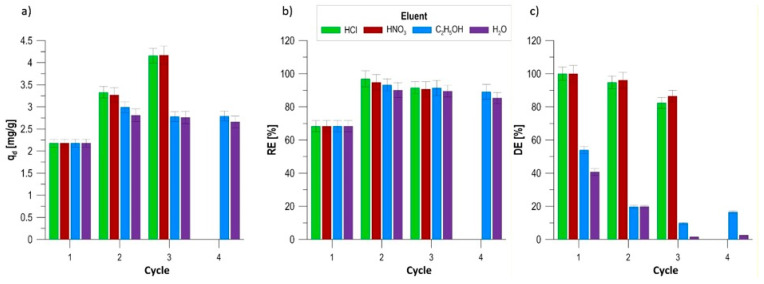
Biocomposite regeneration: (**a**) sorption capacity, (**b**) MB removal rate, and (**c**) MB desorption rate.

Evaluating the potential for multiple sorbent reuse and the efficiency of adsorbed dye recovery is a key parameter determining the application and economic potential of the new material. Methylene blue desorption processes were analyzed in a multi-cycle system using various eluents: mineral acids (0.1 mol/dm^3^ HNO_3_ and HCl), ethyl alcohol (48% C_2_H_5_OH), and distilled water (Figure 9). The highest desorption efficiency (DE) was observed using mineral acids, which is directly correlated with the electrostatic properties of the biocomposite surface identified during the pH_PZC_ study [51]. Nitric acid proved to be the most effective eluent, achieving DE = 100% in the first cycle, which remained high in subsequent phases (96.1% in the second cycle and 86.5% in the third cycle). Hydrochloric acid demonstrated similar characteristics, with full dye recovery in the first cycle and a gradual decrease to 82.4% in the third regeneration process. The mechanism of such efficient desorption in an acidic environment is based on the rapid protonation of the biocomposite’s functional groups. High H^+^ ion concentrations lead to a positive surface charge, which generates strong electrostatic repulsion forces between the matrix and the methylene blue cations, forcing them into solution [51]. Despite the high desorption efficiency, the use of strong mineral acids was associated with progressive degradation of the biocomposite matrix. After only the second regeneration cycle in HNO_3_ and HCl, degradation processes were observed, manifesting as a steady loss of material mass and a change in its morphology. This phenomenon forced the discontinuation of tests in these media after the third cycle. The next step involved regeneration tests using environmentally benign eluents. Biocomposites regenerated using a 48% ethanol solution and distilled water demonstrated significantly higher mechanical stability. This allowed for four complete sorption–desorption cycles to be conducted without any visible signs of matrix degradation. Desorption efficiency in ethanol and water was significantly lower (DE_ethanol_ = 54.0–9.8%; DE_water_ = 40.7–1.5%), confirming that dye binding is strong and relies on mechanisms that are more difficult to reverse under neutral pH conditions. Additionally, an unusual phenomenon of an increase in the amount of sorbed dye with the number of regeneration cycles was observed. For example, after regeneration in HCl, the sorption capacity increased from the initial level of 2.18 mg/g to 3.33 mg/g in the second cycle and 4.16 mg/g in the third cycle. This is assumed to be the result of partial chemical degradation of the matrix, which paradoxically leads to the exposure of new active sites and an increase in the specific surface area available for the sorbate. The developed biocomposite showed the highest regeneration potential in the environment of 0.1 mol/dm^3^ HNO_3_, however, this process requires optimization to enhance the mechanical stability of the polymer matrix to enable operation in a larger number of cycles without losing the material integrity [52].

It should be noted that while mineral acids (HNO_3_ and HCl) provided the highest desorption efficiencies, their use may be less consistent with the green and sustainable character of the proposed biocomposite. In this study, acidic eluents were therefore applied primarily as reference reagents to determine the maximum regeneration potential and to elucidate the binding strength of methylene blue to the sorbent. From an environmental and practical perspective, milder regeneration strategies, such as alcohol-based eluents, pH-controlled desorption, or the use of dilute salt solutions, are more attractive alternatives, even if they result in lower desorption efficiencies. Notably, regeneration with ethanol and water allowed for repeated reuse of the biocomposite without significant structural degradation, indicating that environmentally benign regeneration methods may be preferable for long-term application of the material.

### 3.9. Biocomposite Characteristics

**Figure 10:**
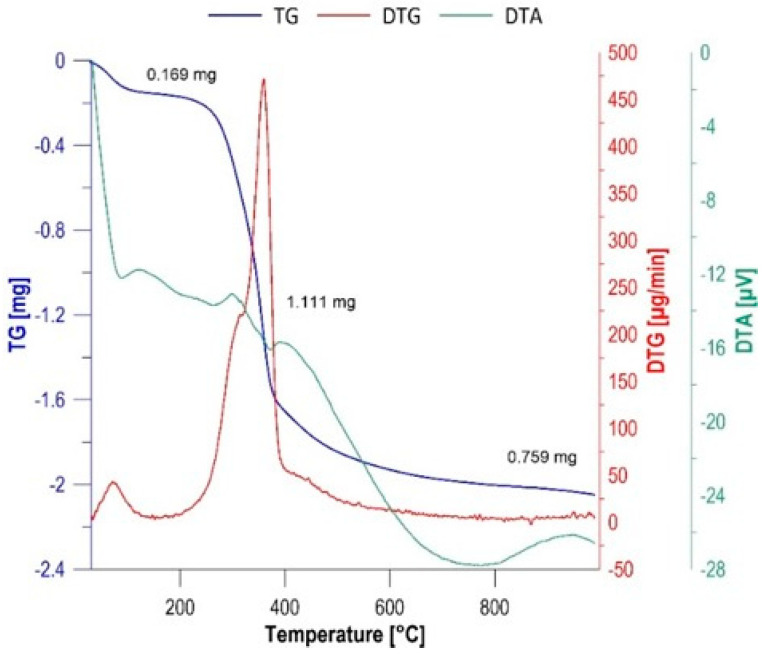
Thermal analysis of aspen wood.

Thermogravimetric (TGA) and differential thermogravimetry (DTG) studies of aspen wood chips allowed for precise determination of the thermal degradation profile of the starting biomass, which served as a criterion for selecting optimal pyrolysis parameters. Recording mass loss as a function of temperature (Figure 10) revealed a three-stage decomposition mechanism characteristic of complex lignocellulosic materials. In the first stage (30–150 °C), a 5.36% mass loss was recorded. This process is primarily associated with the endothermic evaporation of hygroscopic water and water physically bound in the wood capillaries. This stage also involves the desorption of low-molecular-weight volatile organic compounds, preparing the polymer structure for actual pyrolysis. The second stage, the main thermal degradation (150–500 °C), is the key phase of the process, where the most rapid mass loss was recorded, reaching 35.05%. From a scientific perspective, this stage corresponds to the thermal depolymerization and fragmentation of the main components of cell walls: hemicellulose and cellulose. Hemicellulose, due to its branched and amorphous structure, decomposes at a lower temperature range (approximately 200–300 °C). Cellulose, characterized by a higher degree of crystallinity, degrades at higher temperatures (above 300 °C), leading to the formation of a carbon-rich aromatic skeleton. The third stage is carbonization and stabilization (500–1000 °C) [53]. The final phase is characterized by a significant slowdown in the rate of mass loss, which reached 23.9%. In this temperature range, pyrolysis of the most stable component, lignin, occurs, which has an extensive, cross-linked phenolic structure. Secondary reactions, such as autogasification and the condensation of aromatic rings, also occur here, leading to the final formation of the porous structure of biochar. Based on the obtained thermograms, it was determined that 550 °C is the optimal temperature for the pyrolysis process. This choice is justified by the completion of the main stage of lignocellulosic fraction decomposition, which ensures obtaining a material with high chemical stability while maintaining a sufficient number of functional groups necessary for biosorption processes [54].

**Figure 11:**
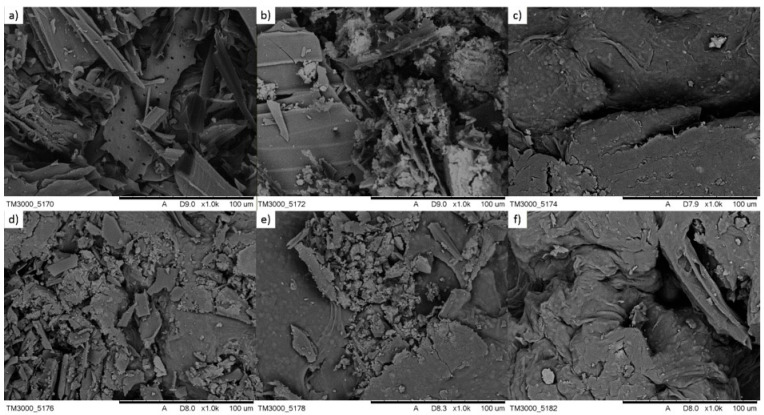
SEM image: (**a**) biochar, (**b**) biochar + magnetite, (**c**) biocomposite, (**d**) biocomposite after sorption, (**e**) biocomposite after regeneration with HNO_3_, and (**f**) biocomposite after regeneration with C_2_H_5_OH.

Scanning electron microscopy (SEM) was used to visualize the surface structure of the biocomposite and assess the degree of immobilization of yeast biomass on the biochar-alginate matrix. Analysis of the micrographs allows for a direct correlation between the material’s porosity and its high sorption capacity, determined using nonlinear models (D–R model, q_d_ = 39.308 mg/g). SEM images revealed a highly developed, heterogeneous biocomposite surface, justifying the high fit of the Temkin and Freundlich isotherm model. The first stage of morphological analysis involved evaluation of the pure carbon support (Figure 11a). The micrographs revealed a porous carbon skeleton formed by the pyrolysis of aspen chips at 550 °C. This process, previously monitored by TGA analysis, led to the removal of volatile fractions and the formation of regular capillary systems and channels of varying diameters (micro- and macropores), which constitute the primary scaffold for the active components [55]. The next step in the synthesis was the introduction of a magnetic phase. SEM images of biochar with magnetite (Figure 11b) revealed the presence of fine, crystalline iron oxide deposits uniformly encrusting the carbon pore walls. The presence of these structures gives the material superparamagnetic properties without completely blocking the lumen of the macropores, which is crucial for maintaining a high specific surface area [56]. The alginate matrix then forms an irregular, folded layer on the biochar surface, acting as a structural binder. In the finished biocomposite (Figure 11c), the surface becomes more compact and “saturated” with polymer. It increases the accessibility of negatively charged carboxyl groups, which is crucial for binding the cationic dye via electrostatic interactions. A key element of SEM imaging is the confirmation of the presence of yeast cells within the composite structure. Spherical and ellipsoidal structures with a diameter of several micrometers, corresponding to *Phaffia rhodozyma* cells, are visible on the surface and in the matrix cavities. Their permanent immobilization is the result of alginate cross-linking, which prevents the leaching of biomass during sorption–desorption processes [57]. SEM images after the sorption process (Figure 11d) reveal the presence of fine deposits on the cell surface and within the pores of the biochar. This suggests a cooperative mechanism in which methylene blue binds not only to the external surface (physical adsorption), but also penetrates biological structures (biosorption). The surface roughness and the presence of numerous edges visible in the SEM images explain the high 1/n constant in the Freundlich model (1/n > 2), which indicates cooperative adsorption on energetically heterogeneous sites. Analysis of the material’s morphology after the regeneration process provided significant information. Micrographs of the sorbent after rinsing with mineral acids (e.g., HNO_3_) revealed distinct structural changes (Figure 11e). The aggressive action of the eluent led to partial chemical degradation of the alginate matrix, which manifested itself in increased edge fragility and local exposure of the biochar structures [58]. Paradoxically, this partial “etching” of the polymer can unclog previously blocked micropores, which was confirmed by experimental results demonstrating an increase in sorption capacity in subsequent regeneration cycles. The dense, porous network visible at the microscale affects dye regeneration, explaining why mild eluents (water, ethanol) exhibit low desorption efficiency (below 55%) (Figure 11f). The integrity of the biochar–alginate interface observed in SEM confirms the amorphous nature of the structure revealed by XRD studies, which provides the matrix flexibility necessary during pH changes during regeneration cycles. XRD analysis confirmed the presence of crystalline magnetite (Fe_3_O_4_) in the biocomposite, while the remaining components exhibited predominantly amorphous character associated with the alginate matrix and immobilized yeast biomass, as shown in Figure S1. SEM analysis provides visual evidence of the successful synthesis of the ternary biocomposite. The synergy between microporous biochar (providing high specific surface area), alginate (providing ion exchange sites), and yeast (offering active biosorption groups) creates a unique sorbent architecture. This morphological structure is the direct cause of achieving equilibrium within a short time (3 h) and the high thermal and crystallographic stability of the material.

**Figure 12:**
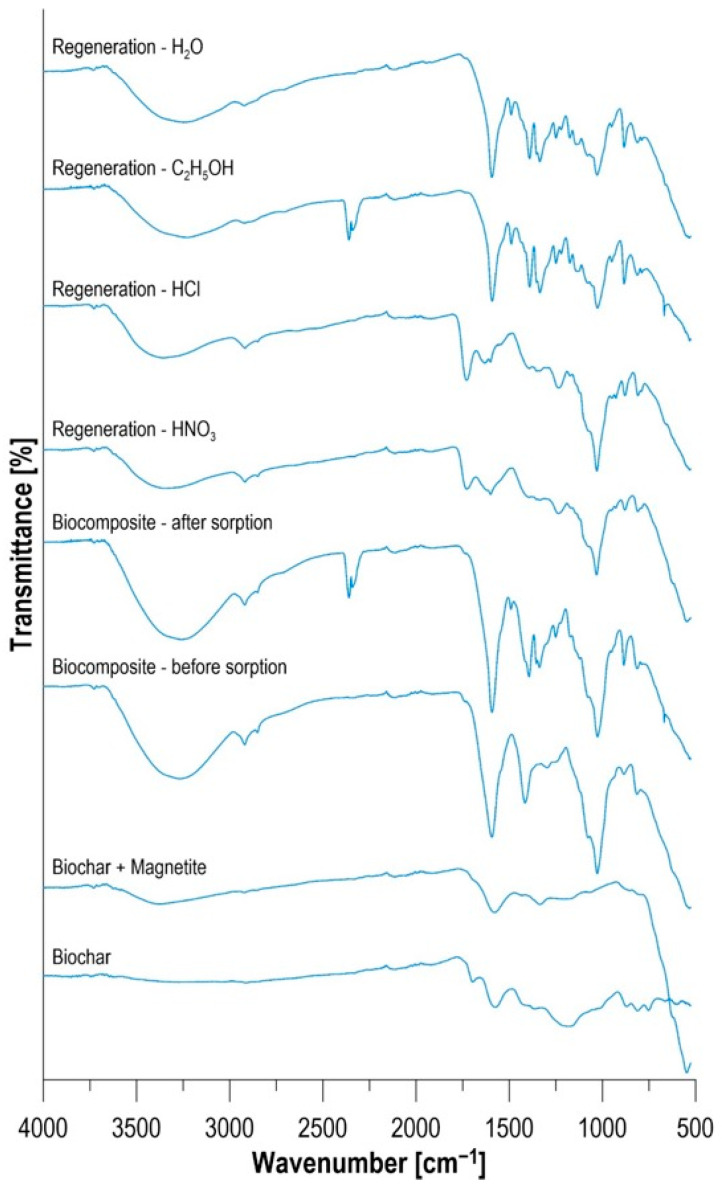
FTIR spectra.

Collective analysis of FTIR spectra (Figure 12) allowed for the precise tracking of changes in the functional group structure at each stage of the biocomposite life cycle. The signals recorded for pure biochar (BC), magnetic biochar (MBC), the finished sorbent, and samples after sorption and desorption provide chemical evidence of the hybrid nature of the material and the reversibility of surface processes. The evolution of the spectra during the biocomposite synthesis process confirms the successful incorporation of subsequent components (biochar → biochar + magnetite → biocomposite). Compared to pure biochar, the biochar + magnetite spectrum displayed a characteristic, sharp band in the 550–590 cm^−1^ range. This band corresponds to stretching vibrations of the Fe–O bonds in the octahedral and tetrahedral structures of magnetite (Fe_3_O_4_) [59]. This confirms that the magnetization process is not merely superficial but leads to the permanent binding of iron oxides to the carbon skeleton. The transition to a full biocomposite is manifested by a rapid increase in band intensity in two regions. A broad band in the 3200–3500 cm^−1^ range (–OH stretching vibrations) indicates the incorporation of numerous hydroxyl groups from alginate and the polysaccharide structures of the yeast *P. rhodozyma*. The most important change is the appearance of a pair of intense bands at approximately 1590 cm^−1^ and 1414 cm^−1^, which are signatures of the asymmetric and symmetric stretching vibrations of carboxyl groups (–COO^−^), respectively [60]. Comparison of the biocomposite spectrum before and after dye sorption allows for conclusions regarding the nature of adsorbate–adsorbent interactions. After contact with MB, a distinct red shift in the carboxyl group bands was observed. The change in the signal level at 1590 cm^−1^ suggests that the negatively charged –COO^−^ centers of the alginate matrix act as the primary binding sites for cationic methylene blue molecules. This confirms the ion-exchange mechanism. Visible changes in the band intensity in the 1500–1600 cm^−1^ range (skeletal vibrations of the C=C rings) and the appearance of subtle signals in the “fingerprint” region (below 1000 cm^−1^) indicate the presence of π–π stacking interactions between the aromatic dye systems and the graphene-like biochar layers [60]. This explains the high efficiency of the material at low sorbate concentrations. Analysis of the spectra after regeneration with four eluents provides key information on the chemical stability of the matrix. After acid regeneration (0.1 M HNO_3_ and HCl), the spectra showed the most dramatic changes. The carboxylic salt (–COO^−^) bands almost completely disappeared in favor of a new band at approximately 1715–1730 cm^−1^, characteristic of protonated acid groups (–COOH) [61]. This process, although ensuring 100% desorption efficiency (through displacement of MB^+^ cations by H^+^ ions), leads to destabilization of the matrix cross-links, which is manifested by the blurring of the bands in the 1000–1100 cm^−1^ range (C–O bonds). The spectra after regeneration in water and ethanol (H_2_O and 48% C_2_H_5_OH) showed a high degree of convergence with the spectrum of the starting material. The preservation of the intensity and position of the COO^−^ bands proves that the chemical structure of the biocomposite does not degrade in these media. The lack of a significant MB signal in the spectra of these samples (despite the low desorption efficiency) suggests that the dye remains strongly bound in the internal pores of the material, which is consistent with the multicycle results. The FTIR composite clearly confirms that the developed biocomposite is a multifunctional material. The high dye removal efficiency results from the synergy of carboxyl groups (alginate), hydroxyl groups (biomass), and aromatic active centers (biochar). Spectroscopic analysis also explains the cause of the material’s physicochemical degradation in an acidic environment through the conversion of the salt form to the acid form, which destroys the polymer network, which is crucial information when designing continuous water treatment systems.

A key functional aspect of the developed material is its ability to rapidly separate from the aqueous phase after the sorption process is complete. For this purpose, the magnetic properties of the biocomposite were analyzed using vibrational magnetometry (VSM) at 25 °C, and the results are presented as a hysteresis loop in Figure S2. Analysis of magnetization in an external magnetic field with an induction of 1.5 T allowed for the determination of key material parameters. The value for the finished biocomposite was 9.3 A·m^2^/kg. Compared to the literature, the magnetization level of the tested material was ten times lower than the values characteristic for pure magnetite (92 A·m^2^/kg) [62]. This decrease is scientifically justified by the so-called matrix effect. The magnetic phase (magnetic biochar) constitutes only a portion of the total mass of the composite, which consists primarily of diamagnetic and nonmagnetic components: sodium alginate and *Phaffia rhodozyma* yeast biomass [63]. The proportional loss of saturation magnetization confirms the effective and permanent “entrapment” of magnetic particles within the biopolymer structure. The shape of the recorded hysteresis loop, characterized by negligible coercivity and remanence, indicates superparamagnetic behavior of the material, which is typical for iron oxide nanoparticles below the single-domain size limit. A very low coercivity (H_c_) and minimal residual magnetism (remanence) are critical parameters for sorption applications. Thanks to low remanence, the biocomposite particles do not exhibit permanent magnetic moments after the external field is removed. This prevents their undesirable clumping in solution, maintaining a high specific surface area available for methylene blue in subsequent cycles [64]. Despite the approximately tenfold lower M_s_ value compared to pure Fe_3_O_4_, the obtained level of 9.3 A·m^2^/kg is fully sufficient to generate a tractive force enabling complete separation of the sorbent from the sorbate solution using standard neodymium magnets.

## 4. Conclusions

The study demonstrated that the developed magnetic biocomposite based on aspen biochar, sodium alginate, and *Phaffia rhodozyma* yeast biomass constitutes a highly effective and stable sorbent for the removal of methylene blue from aqueous solutions. Structural analysis using FTIR and XRD confirmed the successful immobilization of the components in the amorphous polymer matrix and the presence of key functional groups (carboxyl and hydroxyl), which constitute the main active sites of the biosorption process. The dye binding mechanism was demonstrated to be physicochemical in nature and proceeds in two stages: the initial, rapid surface adsorption on the biochar skeleton was followed by slower biosorption and intrapore diffusion into the yeast cellular structures, resulting in the establishment of dynamic equilibrium after 180 min of contact. The use of two time regimes in the equilibrium studies (3 h and 24 h) allowed for the demonstration of a progressive increase in sorption capacity with increasing contact time, which is associated with the metabolic and structural activity of yeast biomass. The theoretical maximum sorption capacity determined from the Dubinin–Radushkevich model (q_d_) was 39.31 mg/g, which, given the high fit of the results to the Freundlich and Temkin models (R^2^ > 0.99), indicates energetic heterogeneity of the surface and a significant contribution of electrostatic interactions. The significant influence of the biological component on the process efficiency was confirmed in thermal studies, where a decrease in sorption efficiency was observed at 35 °C compared to the optimal conditions established at 25 °C (q_e_ = 1.43 mg/g). The composite’s magnetic properties, characterized by a saturation magnetization of 9.3 A·m^2^/kg and a superparamagnetic hysteresis loop, enable complete phase separation of the material in less than 30 s using an external magnetic field, eliminating the need for cumbersome filtration. Sorbent regeneration studies have shown that despite the complete desorption of methylene blue in a mineral acid environment, the most beneficial solution for maintaining the structural integrity of the matrix is the use of alcohol eluents, which allow for stable operation of the biocomposite through at least four sorption–desorption cycles.

## Figures and Tables

**Table 1:** Parameters of kinetic models for MB adsorption on the biocomposite.

Kinetic Model	Parameters			
Pseudo-first order rate	ARE [%]	R^2^	q_1_ [mg/g]	k_1_ [1/min]
	3.86	0.9966	2.3617355	2.3855003
Pseudo-second order rate	ARE [%]	R^2^	q_2_ [mg/g]	k_2_ [g/(mg·min)]
	4.78	0.9906	2.550	1.343
Elovich	ARE [%]	R^2^	α [mg/(g·min)]	β [g/mg]
	18.75	0.8979	49.723	2.793
Weber–Morris	ARE [%]	R^2^	I	K_id_
	37.71	0.6421	1.3825	0.3174

**Table 2:** Adsorption isotherm parameters for the adsorption of MB.

Isotherm Model	Parameters				
Langmuir		ARE [%]	R^2^	q_m_ [mg/g]	K_L_ [dm^3^/mg]
	3 h	17.14	0.8766	n.d.	n.d.
	24 h	25.31	0.8281	n.d.	n.d.
Freundlich		ARE [%]	R^2^	K_F_ (mg^1 − (1/n)^(dm^3^)^1/n^g^−1^)	1/n
	3 h	4.10	0.9929	0.00348	1.937
	24 h	8.39	0.9904	0.00229	2.267
Temkin		ARE [%]	R^2^	K_T_ [dm^3^/g]	B [kJ/mol]
	3 h	1.59	0.9984	0.04950	6.567
	24 h	7.45	0.9967	0.06123	8.406
D–R		ARE [%]	R^2^	K_ad_ [mol^2^/kJ^2^]	q_d_ [mg/g]
	3 h	2.02	0.9983	0.02876	25.216
	24 h	7.96	0.9966	0.02546	39.308

n.d. (not determined)—The parameters of the Langmuir model were not determined due to the lack of model convergence and the obtaining of physically unrealistic values.

## Data Availability

The original contributions presented in this study are included in the article/supplementary material. Further inquiries can be directed to the corresponding author.

## Supplementary Materials

The following supporting information can be downloaded at: https://www.mdpi.com/article/10.3390/ma19091894/s1, Table S1: Equations used in calculations; Table S2: Results of the study on the selection of the appropriate mass fraction of biochar; Table S3: Results obtained during 1 hour of testing for the selection of the most favorable mass fraction of inoculum; Table S4: Results obtained during a 24-hour study of selecting the most favorable mass fraction of inoculum; Table S5: Results obtained during a 1-hour study determining the effect of microorganism centrifugation on sorption; Table S6: Results obtained during a 24-hour study determining the effect of centrifugation of microorganisms on sorption; Figure S1: XRD of biocomposite; Figure S2: VSM of biocomposite.
