# Supplementary material for: Magnetic Biocomposite Based on Aspen Biochar, Sodium Alginate, and Phaffia rhodozyma Yeast for Efficient Removal of Methylene Blue from Aqueous Solutions
Source: Materials (Basel). 2026 May 4;19(9):1894. doi: 10.3390/ma19091894 (PMC13165108; doi:10.3390/ma19091894)
Supplement: Supplementary file 1 [file materials-19-01894-s001.zip › materials-4291063-supplementary.pdf]

Table S1. Equations used in calculations.

No.		Equations
**1**	The sorption capacity	$q_e = \frac{(c_0 - c_e)V}{m}$
**2**	The percentage removal at the equilibrium	$RE = \frac{c_0 - c_e}{c_0} 100\%$
**3**	Langmuir model	$q_e = \frac{q_m K_L C_e}{1 + K_L C_e}$
**4**	Freundlich model	$q_e = K_F C_e^{1/n}$
**5**	Temkin model	$q_e = B \ln K_T C_e$
**6**	Dubinin–Radushkevich model	$q_e = q_d \exp(-K_{ad} \varepsilon^2)$<br>$\varepsilon = RT \ln\left(1 + \frac{1}{C_e}\right)$
**7**	Pseudo-first order model	$q = q_e(1 - \exp(-k_1 t))$
**8**	Pseudo-second order model	$q_t = \frac{t}{(1/k_2 q_{e)}^2 + (t/q_e)}$
**9**	Elovich model	$q_t = \frac{1}{\beta} \ln(1 + \alpha\beta t)$
**10**	Weber–Morris model	$q_t = K_{id}\sqrt{t} + I$
**11**	The coefficient of determination	$R^2 = 1 - \frac{\sum_1^n (q_{exp} - q_{pred})^2}{\sum_1^n (q_{exp} - \overline{q_{exp}})^2}$
**12**	Average relative error	$ARE = \frac{100}{n} \sum^n \frac{\lvert q_{exp} - q_{pred} \rvert}{q_{exp}}$
**13**	The desorption efficiency	$DE = \frac{C_d \cdot V_d}{q_d \cdot m} \cdot 100\%$

Table S2. Results of the study on the selection of the appropriate mass fraction of biochar.

VARIANT	q_e [mg/g]	RE [%]
**1**	2.64	28.7
**2**	2.62	27.3

**3**	2.30	25.2
**4**	1.98	21.1

Table S3. Results obtained during 1 hour of testing for the selection of the most favorable mass fraction of inoculum.

**VARIANT**	**q_e [mg/g]**	**RE [%]**
**1**	0.761	37.6
**2**	0.815	40.4
**3**	0.769	38.2
**4**	0.723	35.9

Table S4. Results obtained during a 24-hour study of selecting the most favorable mass fraction of inoculum.

**VARIANT**	**q_e [mg/g]**	**RE [%]**
**1**	1.02	50.7
**2**	1.17	58.2
**3**	1.13	56.0
**4**	1.04	51.8

Table S5. Results obtained during a 1-hour study determining the effect of microorganism centrifugation on sorption.

**VARIANT**	**q_e [mg/g]**	**RE [%]**
**1**	1.76	37.1
**2**	1.89	39.8
**3**	1.50	31.7

Table S6. Results obtained during a 24-hour study determining the effect of microorganism centrifugation on sorption.

VARIANT	q_e [mg/g]	RE [%]
**1**	2.66	56.3
**2**	2.96	62.8
**3**	2.35	49.7

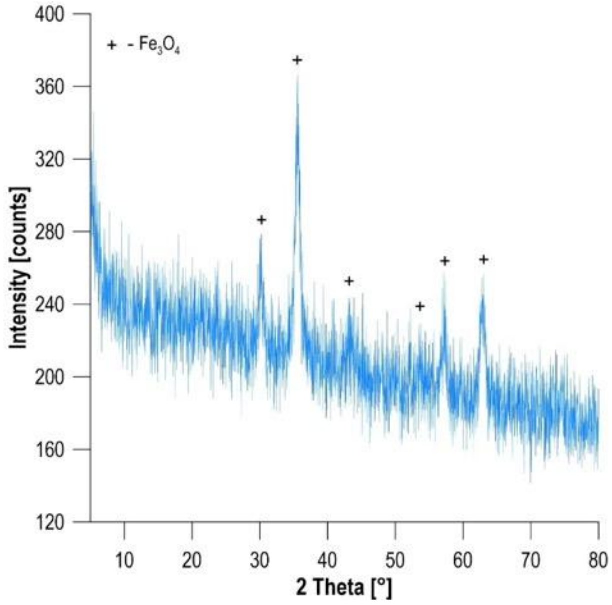

Figure S1. XRD of the biocomposite.

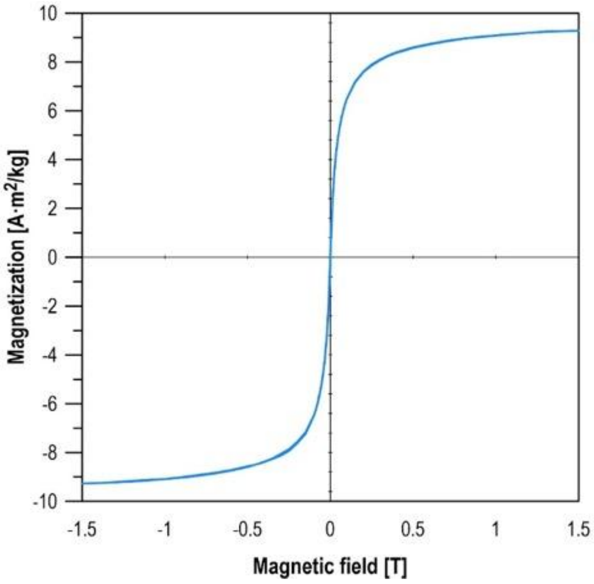

Figure S2. VSM of the biocomposite.
